# Supplementary material for: Transcriptome Analysis and Functional Characterization Reveal That Peclg Gene Contributes to the Virulence of Penicillium expansum on Apple Fruits
Source: Foods. 2023 Jan 19;12(3):479. doi: 10.3390/foods12030479 (PMC9914705; doi:10.3390/foods12030479)
Supplement: Supplementary file 1 [file foods-12-00479-s001.zip › Figure S1~S3.pdf]

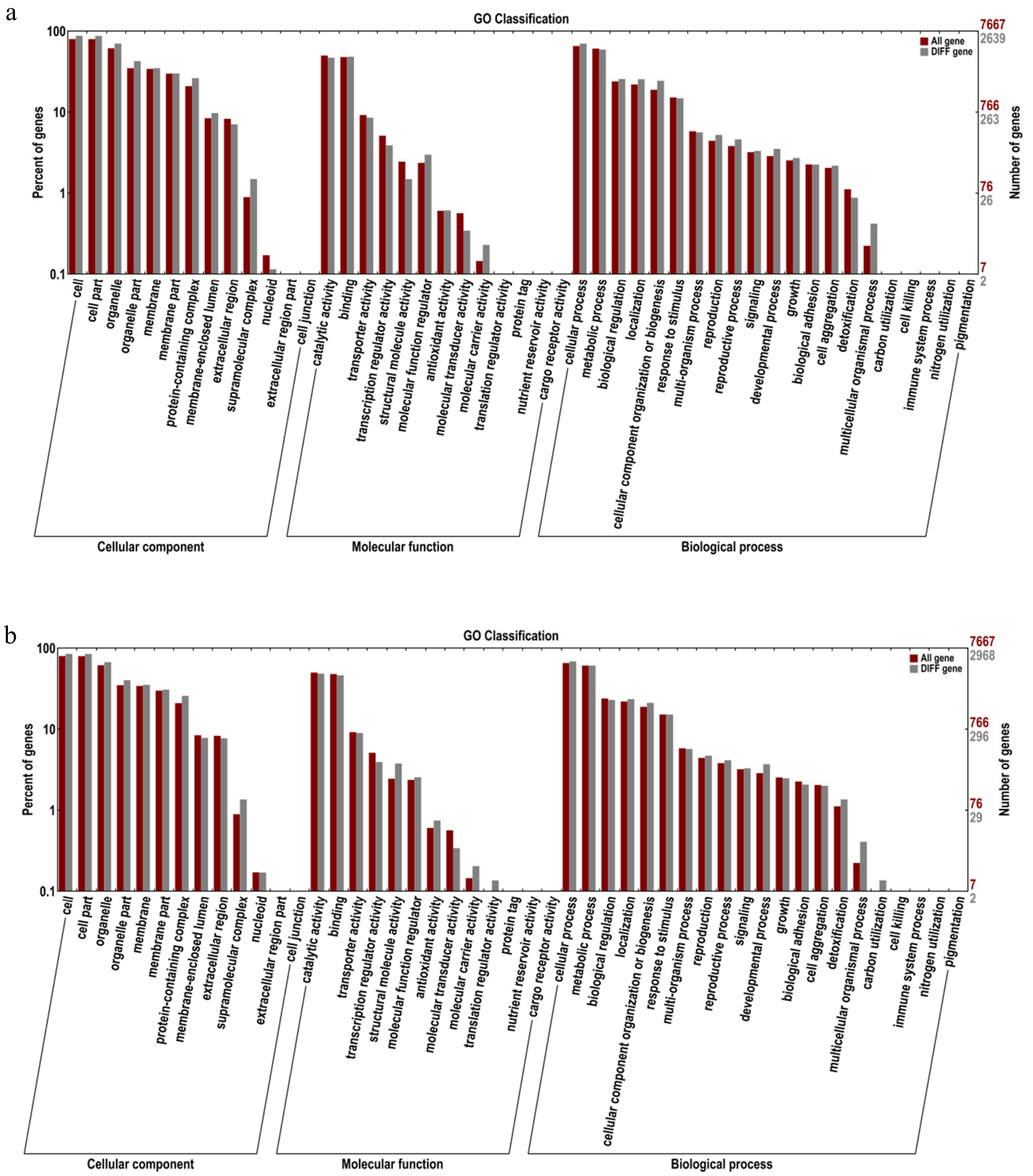

Figure S1. Gene ontology (GO) categorization of DEGs in *P. expansum* during the early stage of infection on apple fruits. (a) 6 hai and (b) 9 hai. GO analysis was performed for three main categories: cellular component, molecular function and biological process.

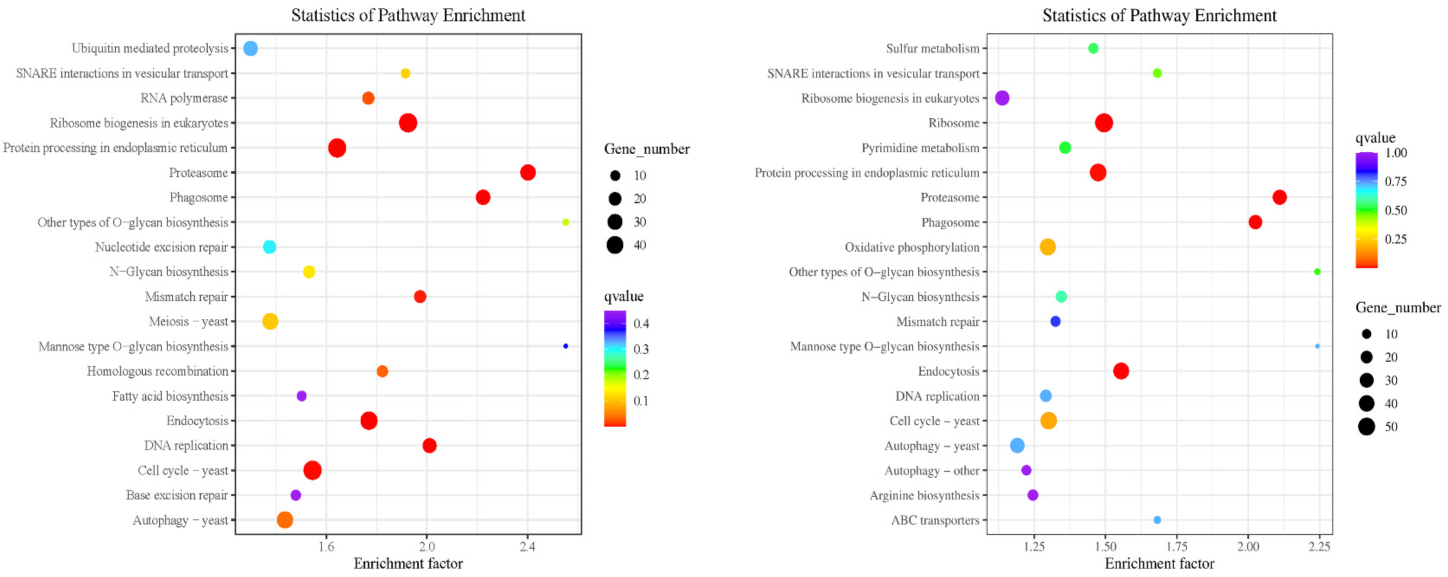

Figure S2. Statistics of Kyoto Encyclopedia of Genes and Genomes (KEGG) pathway enrichment analysis of DEGs in *P. expansum* during the early stage of infection on apple fruits. (a) 6 hai and (b) 9 hai. KEGG pathways are plotted on the ordinate, and the enrichment factor (rich factor) is plotted on the abscissa. The size of points represents the number of DEGs mapped to the reference pathway, and the color of points represents the q-value.

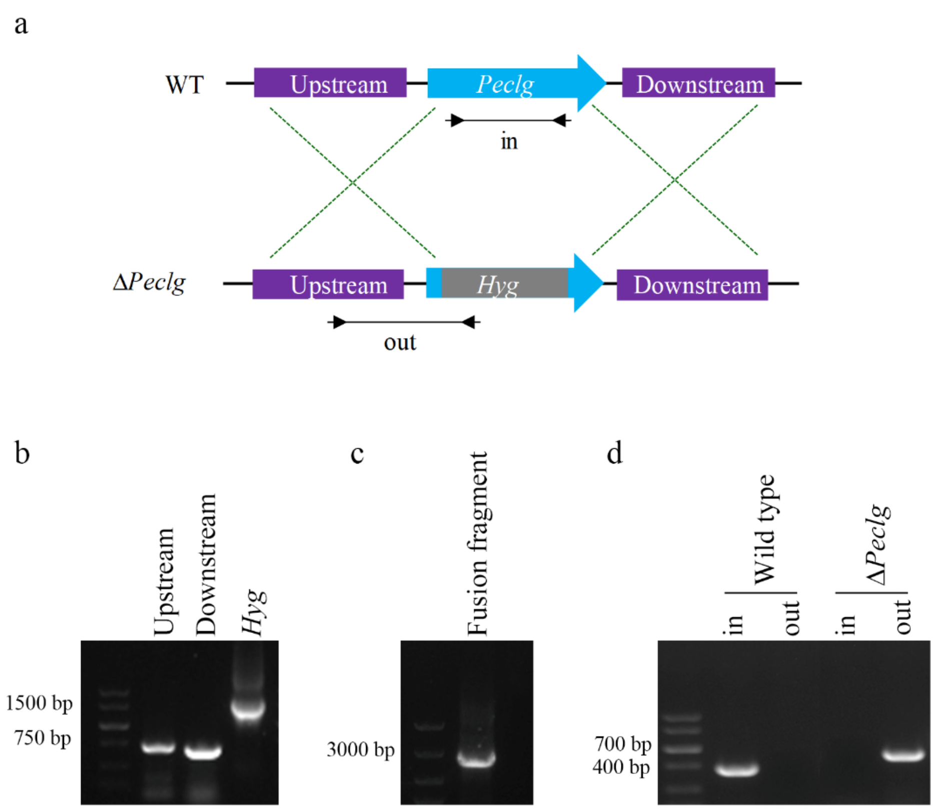

Figure S3. Construction and identification of Δ*Peclg* mutant. (a) Schematic diagram of *Peclg* knockout strategy. (b) Amplification of upstream, downstream and *hyg* fragments. (c) Fusion fragment obtained by overlap PCR amplification. (d) Verification of Δ*Peclg* by PCR amplification using in and out primer pairs.
